# Chronic pain after breast surgery: incidence, associated factors, and impact on quality of life, an observational prospective study

**DOI:** 10.1186/s13741-021-00176-6

**Published:** 2021-02-24

**Authors:** Gianluca Villa, Raffaele Mandarano, Caterina Scirè-Calabrisotto, Valeria Rizzelli, Martina Del Duca, Diego Pomarè Montin, Laura Paparella, A. Raffaele De Gaudio, Stefano Romagnoli

**Affiliations:** 1 Department of Health Sciences, Section of Anaesthesiology, Intensive Care and Pain Medicine, University of Florence, Viale Pieraccini, 6, 50139 Florence, Italy; 2 Department of Anaesthesia and Intensive Care, Azienda Ospedaliero Universitaria Careggi, Largo Brambilla,3, Florence, 50100 Italy

**Keywords:** IASP definition, Axillary surgery, Breast cancer, Numerical rating scale, Brief pain inventory questionnaire

## Abstract

**Background:**

Chronic pain after breast surgery (CPBS) has a disabling impact on postoperative health status. Mainly because of the lack of a clear definition, inconsistency does exist in the literature concerning both the actual incidence and the risk factors associated to CPBS. The aim of this prospective, observational study is to describe the incidence of and risk factors for CPBS, according to the definition provided by the IASP taskforce. The impact of CPBS on patients’ function and quality of life is also described.

**Methods:**

Women aged 18+ undergoing oncological or reconstructive breast surgery from Jan until Apr 2018 at the Breast Unit of Careggi Hospital (Florence, Italy) were prospectively observed. Postoperative pain was measured at 0 h, 3 h, 6 h, 12 h, 24 h, 48 h, and 3 months (CPBS) after surgery. Preoperative, intraoperative, and postoperative factors were compared in CPBS and No-CPBS groups through multivariate logistic regression analysis.

**Results:**

Among the 307 patients considered in this study, the incidence of CPBS was 28% [95% CI 23.1–33.4%]. Results from the logistic regression analysis suggest that axillary surgery (OR [95% CI], 2.99 [1.13–7.87], *p* = 0.03), preoperative use of pain medications (OR [95% CI], 2.04 [1.20–3.46], *p* = 0.01), and higher dynamic NRS values at 6 h postoperatively (OR [95% CI], 1.28 [1.05–1.55], *p* = 0.01) were all independent predictors for CPBS.

**Conclusions:**

Chronic pain after breast surgery is a frequent complication. In our cohort, long-term use of analgesics for pre-existing chronic pain, axillary surgery, and higher dynamic NRS values at 6 h postoperatively were all factors associated with increased risk of developing CPBS. The possibility to early detect persistent pain, particularly in those patients at high risk for CPBS, might help physicians to more effectively prevent pain chronicisation.

**Trial registration:**

ClinicalTrials.gov registration NCT04309929.

**Supplementary Information:**

The online version contains supplementary material available at 10.1186/s13741-021-00176-6.

## Background

Chronic pain after breast surgery (CPBS) is a frequent condition associated with significant morbidity and/or debilitating complications (Odle 2014; Peuckmann et al. 2009). CPBS can range from mild to moderate, with pain lasting for months to years, and can have neuropathic characteristics such as burning, stabbing, pulling, hypoesthesia/anaesthesia, and phantom breast/nipple (Caffo et al. 2003; Jung et al. 2003). Chronic pain has a disabling impact on health status and negatively affects the quality of life of breast cancer survivors (Belfer et al. 2013), as it can affect sleep, work, activities of daily living, and interpersonal relationships (Miaskowski et al. 2012).

The pathophysiology of CPBS is extremely complex and not completely understood (Lavand’homme 2017). Several pathophysiological theories have been postulated to explain the development of chronic pain after surgery, including traumatic nerve injury, inflammation, and peripheral and central sensitisation (Urits et al. 2020). These mechanisms seem particularly pronounced in patients undergoing breast surgery. In particular, female gender seems to be associated with greater pain sensitivity and higher levels of acute and chronic postoperative pain compared to male gender (Deumens et al. 2013). Hormonal characteristics, as well as diverse immune and stress responses to surgery, have been advocated to explain these differences (Deumens et al. 2013). Furthermore, younger patients like those undergoing breast surgery are more prone to robust hormonal and neuroinflammatory responses (Deumens et al. 2013). Finally, breast surgery seems to be associated with a higher incidence and greater severity of nerve injuries compared with other surgical procedures. Because of the complex structure of the axillary and breast regions, nerve severance, compression, ischemia, stretching, and retraction during breast cancer operation, or from subsequent formation of a traumatic neuroma or scar tissue, unlikely preserve neural structures (Sarhadi et al. 1996). All these factors contribute to the higher prevalence of chronic pain in breast surgery patients (Deumens et al. 2013). Based on the pathophysiological mechanisms described above, some have suggested that a multimodal approach—involving, for instance, use of anti-inflammatory drugs (for preventing primary hyperalgesia) or locoregional anaesthesia (nerve blocks or epidural anaesthesia, for preventing secondary hyperalgesia and central nervous system sensitisation)—may limit the transition from acute to chronic pain (Urits et al. 2020).

Several efforts have been made to identify preoperative (Bell et al. 2014; Gartner et al. 2009; Mejdahl et al. 2013; Poleshuck et al. 2006; Spivey et al. 2018; Wang et al. 2018), intraoperative (Mejdahl et al. 2013; Spivey et al. 2018), and postoperative (Fassoulaki et al. 2009; Hamood et al. 2018; Schou Bredal et al. 2014; Wang et al. 2018) factors potentially associated with CPBS, including, but not limited to, young age, genetic features, chemotherapy, radiotherapy, or lymph node dissection. Nonetheless, results are inconsistent, and uncertainty does exist on the definition of those patients susceptible to CPBS. Interestingly, conflicting results are reported in the literature concerning the estimated incidence of CPBS. Although recognised as a frequent complication, the occurrence of CPBS varies across studies ranging from 25 to 60 (Gartner et al. 2009), mainly because of the lack of a clear definition, unequal follow-up time points, and case-mix heterogeneity (Brummett 2011). Only recently, the International Association for the Study of Pain (IASP) Taskforce for the Classification of Chronic Pain has provided a final, unambiguous definition for CPBS, defining it as “pain that develops or worsens after a surgical procedure in the breast area (anterolateral chest wall and, in some cases, the ipsilateral axillary region) and persists at 3 months after surgery” (Schug et al. 2019).

Taking into consideration this new and widely accepted definition, the aim of this prospective, observational study is to describe the incidence of and risk factors for CPBS in a cohort of female patients undergoing surgery for breast cancer. The intensity and impact of CPBS on patients’ daily activities will also be described.

## Methods

All adult (age ≥ 18 years) female patients scheduled for breast surgery from Jan to Apr 2018 at the Breast Unit of Careggi Hospital, a large tertiary care teaching hospital in Florence, Italy, were prospectively observed. The study was approved by the Ethical Committee of Area Vasta Toscana Centro before enrolment of the first patient (N° OSS.16.246, clinicalTrials.gov registration NCT04309929). Each patient gave her consent for participation in this study and publication of the results. Participants were not compensated for their participation in the study.

According to local routine practice, all patients were preoperatively taught how to correctly identify and treat acute postoperative and chronic pain. In particular, the signs and symptoms associated with CPBS (e.g., burning, stabbing, pulling, hypoesthesia/anaesthesia, phantom breast/nipple), and the use of patient-controlled analgesia (PCA) and rescue analgesia were explained to all breast surgery candidates.

Each patient was prospectively observed during the entire perioperative period (from preoperative anaesthesia evaluation to postoperative and post hospital discharge). Demographic characteristics and comorbidities were recorded before surgery, as well as the presence of chronic pain and chronic use of analgesics. Intraoperative surgical and anaesthesiologic variables were prospectively recorded right after the surgical procedure. Finally, use of analgesic drugs was recorded at 0 h, 3 h, 6 h, 12 h, 24 h, and 48 h after surgery. Acute postoperative pain was assessed at the same time points using the numerical rating scale (NRS)—ranging from 0 (no pain) to 10 (worst pain)—both in resting conditions (static pain) and during active ipsilateral arm abduction or cough (dynamic pain). Each patient was re-evaluated at 3 months postoperatively for the presence of signs and symptoms of pain that develops or worsens in the breast area. Using a yes/no question, patients were asked to self-report the presence of pain, and, if possible, evaluate its severity through a scale ranging from zero (no pain) to 10 (worst pain imaginable). According to the IASP definition (Schug et al. 2019), the presence of signs and symptoms of pain at 3 months was considered CPBS.

To reduce assessment biases, an anaesthesiologist not involved in the surgical procedure assessed pain both postoperatively and during follow-up. Preoperative, intraoperative, and postoperative variables statistically associated with CPBS were evaluated. Finally, we assessed pain intensity and pain interference with daily functions in patients with CPBS using the Italian validated version of the Brief Pain Inventory (BPI) questionnaire (Bonezzi et al. 2002; Daut et al. 1983). This is a multidimensional 10-item questionnaire aimed at exploring the impact of pain on each item, with values ranging between 0 and 10 (0 = minimal impact, 10 = invalidating impact). Patients with missing preoperative, intraoperative, and postoperative data were excluded from the analysis.

### Statistical analysis

According to data available in the literature (Vilholm et al. 2008), we calculated the sample size required to expect CPBS in 24% of the subjects included (CI 95% interval width 0.1), with 90% statistical power and 0.05 level of significance.

Continuous variables are expressed as means ± standard deviation or medians and interquartile range, according to data distribution (normality assessed through Shapiro-Wilk test). The CPBS and No-CPBS groups of patients were compared for statistical differences using the unpaired Student’s *t*-test and the Wilcoxon’s test for normally and not normally distributed variables, respectively. Categorical variables are expressed as a percentage; statistical differences between the two groups were evaluated through a Chi-square or Fisher’s exact test. A multivariate logistic regression model was designed considering quantitative and qualitative variables significantly associated with CPBS in the univariate analysis (*p*-value < 0.2). A backward selection procedure based on the AIC was used to select the variables in the final model. Results are expressed in terms of *p*-value, odds ratio (OR), and 95% confidence interval (95% CI). The capability of the final model to predict CPBS at 3 months was assessed with a ROC Analysis and expressed as a ROC-AUC. Statistical analysis was performed using the R© software version 3.5.1.

## Results

**Fig. 1:**
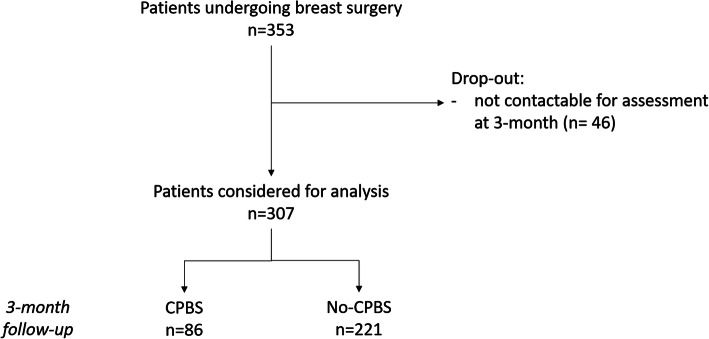
Flow chart of patient enrolment and pain detection

Three hundred and fifty-three patients underwent breast surgery in the study period and were prospectively observed during the perioperative period. Of these, 46 (13%) dropped out of the study because they were not contactable for assessment at 3 months follow-up; 307 patients were thus considered for the analysis (Fig. 1). Patients lost in the follow-up had demographic characteristics and comorbidities not statistically different than those considered for the final analysis.

The incidence of CPBS at 3 months was 28% [95% CI 23.1–33.4%] (median NRS 5 [3–7]). Table 1 displays patients’ baseline characteristics, and surgical and anaesthesiologic factors. Most patients underwent mastectomy or breast conservation surgery, alone or in combination with other surgical procedures (e.g., cosmetic reconstruction and/or axillary surgery), as described in Table 1. All surgical procedures were performed under general anaesthesia alone (total intravenous or inhaled anaesthesia) or in association with other forms of regional anaesthesia (PECS block or local anaesthetics infiltration). A combination of morphine, ketorolac tromethamine, and paracetamol was used for analgesia at the end of surgery.

**Table 1:** Patients’ baseline characteristics, surgical, and anaesthesiologic factors in CPBS and No-CPBS groups

	Total (***n*** = 307)	No-CPBS (***n*** = 221)	CPBS (***n*** = 86)	OR [95% CI]	*p*
**Patient characteristics**					
Age (years)	56.2 ± 12.4	55.3 ± 12.4	53.6 ± 11.2	0.99 [0.97–1.01]	0.275
Height (cm)	163.5 ± 6.3	163.6 ± 6.6	163.4 ± 5.3	0.99 [0.96–1.04]	0.824
Weight (kg)	64.5 ± 12.5	55.3 ± 5.9	55.2 ± 4.8	0.10 [0.99–1.03]	0.212
BMI (Kg/m^2^)	24.1 ± 4.4	23.9 ± 4.1	24.7 ± 4.9	1.04 [0.98–1.10]	0.168
Preoperative pain	95 (30.9%)	56 (25.3%)	39 (45.3%)	1.13 [0.64–1.97]	0.686
Chronic use of pain drugs	19 (6.2%)	9 (4.1%)	10 (11.6%)	3.10 [1.21–7.92]	0.018
Anxiety/depression	43 (14%)	33 (14.9%)	10 (11.6%)	0.75 [0.35–1.60]	0.446
Fibromyalgia	2 (0.7%)	1 (0.5%)	1 (1.2%)	2.59 [0.16–41.90]	0.511
Neoadjuvant-CHT	23 (7.5%)	15 (6.8%)	8 (9.3%)	1.41 [0.58–3.45]	0.461
Preop. hormone therapy	37 (12.1%)	28 (12.7%)	9 (10.5%)	0.81 [0.36–1.79]	0.589
Neoadjuvant-RT	1 (0.33%)	1 (0.5%)	0 (0.0%)	–	–
Previous ipsilateral breast surgery	108 (35.18%)	78 (35.3%)	30 (34.9%)	0.98 [0.58–1.66]	1.000
School education					0.517
Primary school	30 (9.77%)	24 (10.9%)	6 (7.0%)	0.79 [0.28–2.24]	
Secondary school	57 (18.57%)	40 (18.1%)	17 (19.8%)	1.35 [0.62–2.93]	
High school	145 (47.23%)	100 (45.2%)	45 (52.3%)	1.42 [0.75–2.69]	
University	75 (24.43%)	57 (25.8%)	18 (20.9%)	*Ref.*	
**Surgical factors**					
Breast-conserving surgery	149 (48.5%)	110 (49.8%)	39 (45.3%)	0.84 [0.51–1.38]	0.526
Mastectomy	91 (29.6%)	61 (27.6%)	30 (34.9%)	1.41 [0.83–2.39]	0.214
Implant-based reconstructive surgery (tissue-expandersor prosthesis)	113 (36.8%)	81 (36.7%)	32 (37.2%)	1.02 [0.61–1.72]	0.928
Pre-pectoral prosthesis	23 (7.5%)	17 (21.0%)	6 (18.8%)	*Ref.*	–
Under-pectoral prosthesis or tissue-expander	47 (15.3%)	32 (39.5%)	15 (46.9%)	1.33 [0.44–4.05]	0.618
Tissue-expander substitution with prosthesis	43 (14.0%)	32 (39.5%)	11 (34.4%)	0.97 [0.31–3.09]	0.964
Axillary surgery	146 (47.6%)	95 (43.0%)	51 (59.3%)	1.93 [1.17–3.21]	0.011
Sentinel node biopsy	99 (32.3%)	64 (29.0%)	35 (40.7%)	0.94 [0.46–1.96]	0.877
Axillary lymph node dissection	47(15.3%)	31 (14.0%)	16 (18.6%)		
Cosmetic surgery	68 (22.2%)	48 (21.7%)	20 (23.3%)	1.09 [0.60–1.98]	0.762
Dorsal-flap reconstructive surgery	1 (0.3%)	1 (0.5%)	0 (0.0%)	–	–
Bilateral surgery	103 (33.6%)	70 (31.8%)	33 (38.4%)	1.33 [0.79–2.24]	0.276
Selective pectoral nerves dissection	19 (6.2%)	12 (5.5%)	7 (8.1%)	1.53 [0.58–4.02]	0.390
**Anaesthesiologic factors**					
Total intravenous anaesthesia	251 (81.8%)	180 (81.4%)	71 (82.6%)	0.93 [0.48–1.78]	0.821
Propofol	251 (81.8%)	180 (81.4%)	71 (82.6%)	1.08 [0.56–2.07]	0.821
Remifentanil	221 (72.0%)	157 (71.0%)	64 (74.4%)	1.19 [0.67–2.09]	0.554
Sufentanil	29 (9.5%)	22 (10.0%)	7 (8.1%)	0.80 [0.33–1.95]	0.626
Fentanil	14 (4.6%)	9 (4.1%)	5 (5.8%)	1.45 [0.47–4.47]	0.513
Inhaled anaesthesia	56 (18.2%)	41 (18.6%)	15 (17.4%)	1.07 [0.56–2.07]	0.999
Sevorane	40 (13%)	29 (13.1%)	11 (12.8%)	0.88 [0.23–3.32]	0.846
Desflurane	16 (5.2%)	12 (5.4%)	4 (4.7%)	1.14 [0.30–4.29]	0.846
Remifentanil	32 (10.4%)	22 (10.0%)	10 (11.6%)	0.58 [0.16–1.94]	0.384
Fentanil	24 (7.8%)	19 (8.6%)	5 (5.8%)	1.73 [0.50–5.95]	0.384
Regional anaesthesia	66 (21.5%)	53 (24.0%)	13 (15.1%)	0.56 [0.29–1.10]	0.092
PECS 1	28 (9.1%)	22 (10.0%)	6 (7.0%)	0.68 [0.27–1.74]	0.418
PECS 2	17 (5.6%)	12 (5.4%)	5 (5.8%)	1.08 [0.37–3.15]	0.895
Local anaesthetics infiltration	21 (6.8%)	19 (8.6%)	2 (2.3%)	0.25 [0.03–1.09]	0.075
Intraoperative analgesia					
Paracetamol	265 (86.3%)	188 (85.1%)	77 (89.5%)	1.50 [0.69–3.29]	0.309
Ketorolac	49 (16.0%)	33 (14.9%)	16 (18.6%)	1.30 [0.68–2.51]	0.431
Morphine	242 (7.8%)	169 (76.5%)	73 (84.9%)	1.73 [0.89–3.37]	0.108

Among anaesthesiologic factors, specific drugs used for total intravenous anaesthesia and inhaled anaesthesia are described for both CPBS and No-CPBS groups. For patients treated with locoregional anaesthesia, use of pectoralis and serratus plane blocks (PECS1 and PECS2) and of local anaesthetics infiltration is reported. For variables with more than two levels, OR [95% CI] are expressed with respect to the level used as reference (Ref.). Wald test *p*-values are 0.774 for implant-based reconstructive surgery and 0.517 for school education. Among patients who underwent axillary surgery, differences between those treated with sentinel node biopsy and those treated with axillary lymph node dissection were evaluated with a chi-squared test (OR [95% CI] = 0.94 [0.46–1.96], *p*-value = 0.877)

Drugs used for postoperative analgesia are shown in Table 2. About half of the patients were prescribed paracetamol 1 g or 500 mg q.i.d. (according to the patient’s weight) and morphine (administered with PCA set up to deliver a bolus of 1 mg of morphine with a lockout interval of 15 min). The remaining patients were prescribed paracetamol in combination with tramadol or ketorolac tromethamine as rescue analgesic. Static and dynamic NRS values in the CPBS and No-CPBS groups are shown in Table 3, as well as their parameters of association with CPBS at univariate analysis.

**Table 2:** Analgesics used postoperatively

	Total (***n*** = 307)	No-CPBS (***n*** = 221)	CPBS (***n*** = 86)	OR [95% CI]	*p*
**Prescribed for postoperative analgesia**					
Paracetamol	307 (100%)	221 (100%)	86 (100%)	–	–
Morphine (PCA)	142 (46.3%)	101 (45.7%)	41 (47.7%)	1.08 [0.64–1.84]	0.799
Tramadol rescue	95 (30.9%)	62 (28.1%)	33 (38.8%)	1.62 [0.93–2.84]	0.074
Ketorolac rescue	70 (22.8%)	54 (24.4%)	16 (21.1%)	0.83 [0.41–1.60]	0.639
**Actually administered for postoperative analgesia**					
**At 3 h**	**Total (*n*** **= 307)**	**No-CPBS (*n*** **= 221)**	**CPBS (*n*** **= 86)**		
Currently using PCA	142/142 (100%)	101/101 (100%)	41/41 (100%)		
Doses requested	1 [0, 21]	0 [0, 21]	1 [0, 9]	1.05 [0.91–1.21]	0.472
Doses administered	1 [1, 6]	0 [0, 6]	1 [0, 5]	1.05 [0.79–1.39]	0.751
Tramadol rescue	4/95 (4.2%)	3/62 (4.8%)	1/33 (3.0%)	0.67 [0.07–6.72]	0.731
Ketorolac rescue	3/70 (4.3%)	3/54 (5.6%)	0/16 (0%)	–	–
**At 6 h**	**Total (*n*** **= 292)**	**No-CPBS (*n*** **= 208)**	**CPBS (*n*** **= 84)**		
Currently using PCA	139/142 (97.9%)	99/101 (98.0%)	40/41 (97.6%)		
Doses requested	1 [0, 26]	1 [0, 26]	1.50 [0, 15]	1.08 [0.98–1.18]	0.133
Doses administered	1 [0, 11]	1 [0, 11]	2 [0, 10]	1.16 [0.98–1.36]	0.078
Tramadol rescue	1/81 (1.2%)	0 (0%)	1/26 (3.8%)	**–**	**–**
Ketorolac rescue	1/57 (1.8%)	1/46 (2.2%)	0 (0%)	–	–
**At 12 h**	**Total (*n*** **= 254)**	**No-CPBS (*n*** **= 180)**	**CPBS (*n*** **= 74)**		
Currently using PCA	106/142 (74.7%)	76/101 (75.2%)	30/41 (73.2%)		
Doses requested	2 [0, 28]	2 [0, 28]	4 [0, 25]	1.06 [0.99–1.14]	0.119
Doses administered	2 [0,14]	2 [0, 13]	3.50 [0, 14]	1.11 [0.98–1.25]	0.097
Tramadol rescue	2/69 (2.9%)	1/46 (2.2%)	1/23 (4.3%)	2.05 [0.12–34.3]	0.619
Ketorolac rescue	5/54 (9.3%)	4/44 (9.1%)	1/10 (10.0%)	1.11 [0.11–11.2]	0.929
**At 24 h**		**No-CPBS (*n*** **= 111)**	**CPBS (*n*** **= 45)**		
Currently using PCA	47/110 (42.7%)	32/79 (40.5%)	15/31 (48.4%)		
Doses requested	3 [0, 35]	3 [0, 23]	4 [1, 35]	1.05 [0.96–1.15]	0.298
Doses administered	3 [0, 17]	3 [0, 17]	4 [1, 14]	1.06 [0.91–1.24]	0.292
Tramadol rescue	0/26 (0%)	0 (0%)	0 (0%)	–	–
Ketorolac rescue	2/33 (6.1%)	2/27 (7.4%)	0 (0%)	–	–
**At 48 h**		**No-CBPS (*n*** **= 35)**	**CPBS (*n*** **= 23)**		
Currently using PCA	4/142 (2.82%)	1/24 (4.2%)	3/17 (17.6%)		
Doses requested	12 [4, 13]	11	13 [4, 13]	0.92 [0.47–1.82]	0.817
Doses administered	6.5 [4, 11]	11	4 [4, 9]	–	–
Tramadol rescue	0/8 (0%)	0 (0%)	0 (0%)	–	–
Ketorolac rescue	1/9 (11.1%)	0 (0%)	1 (100%)	–	–

At the different time points, the percentages of patients on the surgical ward using PCA and/or tramadol and ketorolac tromethamine as prescribed rescue analgesics are reported over time. Requested and administered doses are expressed as median and minimum-maximum values. Among patients treated with PCA in the No-CPBS and CPBS groups, those receiving less than 3 doses (1 dose = morphine 1 mg) were respectively 94 (93.1%) and 40 (97.6%) in the first 3 h postoperatively and 84 (84.8%) and 31 (77.5%) in the first 6 h postoperatively; those receiving less than 6 doses in the first 12 h postoperatively were 67 (88.2%) and 26 (86.7%), respectively; those receiving less than 10 doses in the first 24 h were 28 (87.5%) and 14 (93.3%), respectively; those receiving less than 15 doses in the first 48 h postoperatively were 1 (100%) and 3 (100%), respectively

**Table 3:** Static and dynamic postoperative NRS in both CPBS and No-CPBS groups. Numeric rating scale (NRS) values are expressed as median and IQR

Pain intensity	Total (***n*** = 307)	No-CPBS (***n*** = 221)	CPBS (***n*** = 86)	OR [95% CI]	*p*
**At 0 h**					
NRS at rest	1 [0, 9]	1 [0, 9]	1 [0, 8]	1.13 [1.00–1.28]	0.075
NRS during movement	1 [0, 10]	1 [0, 10]	2 [0, 9]	1.12 [1.01–1.25]	0.051
**At 3 h**	**Total (*n*** **= 306)**	**No CPBS (*n*** **= 220)**	**CPBS (*n*** **= 86)**		
NRS at rest	1 [0, 7]	1 [0, 5]	1 [0, 7]	1.28 [1.02–1.62]	0.034
NRS during movement	2 [1, 8]	2 [0, 6]	2 [0, 8]	1.31 [1.08–1.59]	0.004
**At 6 h**	**Total (*n*** **= 292)**	**No CPBS (*n*** **= 208)**	**CPBS (*n*** **= 84)**		
NRS at rest	1 [0, 5]	1 [0, 5]	1 [0, 5]	1.24 [0.98–1.56]	0.101
NRS during movement	2 [1, 7]	2 [0, 5]	2 [0, 7]	1.27 [1.06–1.54]	0.041
**At 12 h**	**Total (*n*** **= 254)**	**No CPBS (*n*** **= 180)**	**CPBS (*n*** **= 74)**		
NRS at rest	1 [0, 6]	1 [0, 6]	1 [0, 5]	1.26 [0.99–1.58]	0.036
NRS during movement	2 [0, 7]	2 [0, 6]	2 [0, 7]	1.24 [1.04–1.48]	0.024
**At 24 h**	**Total (*n*** **= 156)**	**No CPBS (*n*** **= 111)**	**CPBS (*n*** **= 45)**		
NRS at rest	1 [0, 5]	1 [0, 4]	1 [0, 5]	1.36 [0.98–1.90]	0.110
NRS during movement	2 [0, 6]	1 [0, 5]	2 [0, 6]	1.35 [1.06–1.71]	0.024
**At 48 h**	**Total (*n*** **= 58)**	**No CPBS (*n*** **= 35)**	**CPBS (*n*** **= 23)**		
NRS at rest	1 [0, 4]	1 [0, 4]	1 [0, 2]	0.86 [0.47–1.56]	0.817
NRS during movement	2 [0, 6]	2 [0, 5]	2 [0, 6]	1.26 [0.90–1.75]	0.279

All variables statistically associated with CPBS with *p*-value < 0.2 in the univariate analysis were considered for multivariate logistic regression analysis. In the final model, variables independently associated with CPBS were axillary surgery (sentinel node biopsy and/or axillary lymph node dissection) (OR [95% CI], 2.99 [1.13–7.87], *p* = 0.03); preoperative use of pain medications (OR [95% CI], 2.04 [1.20–3.46], *p* = 0.01); and higher dynamic NRS values at 6 h postoperatively (OR [95% CI], 1.28 [1.05–1.55] *p* = 0.01). This model was able to identify the development of CPBS at 3 months postoperatively with a ROC-AUC of 0.67 (95% CI [0.61–0.74]).

**Fig. 2:**
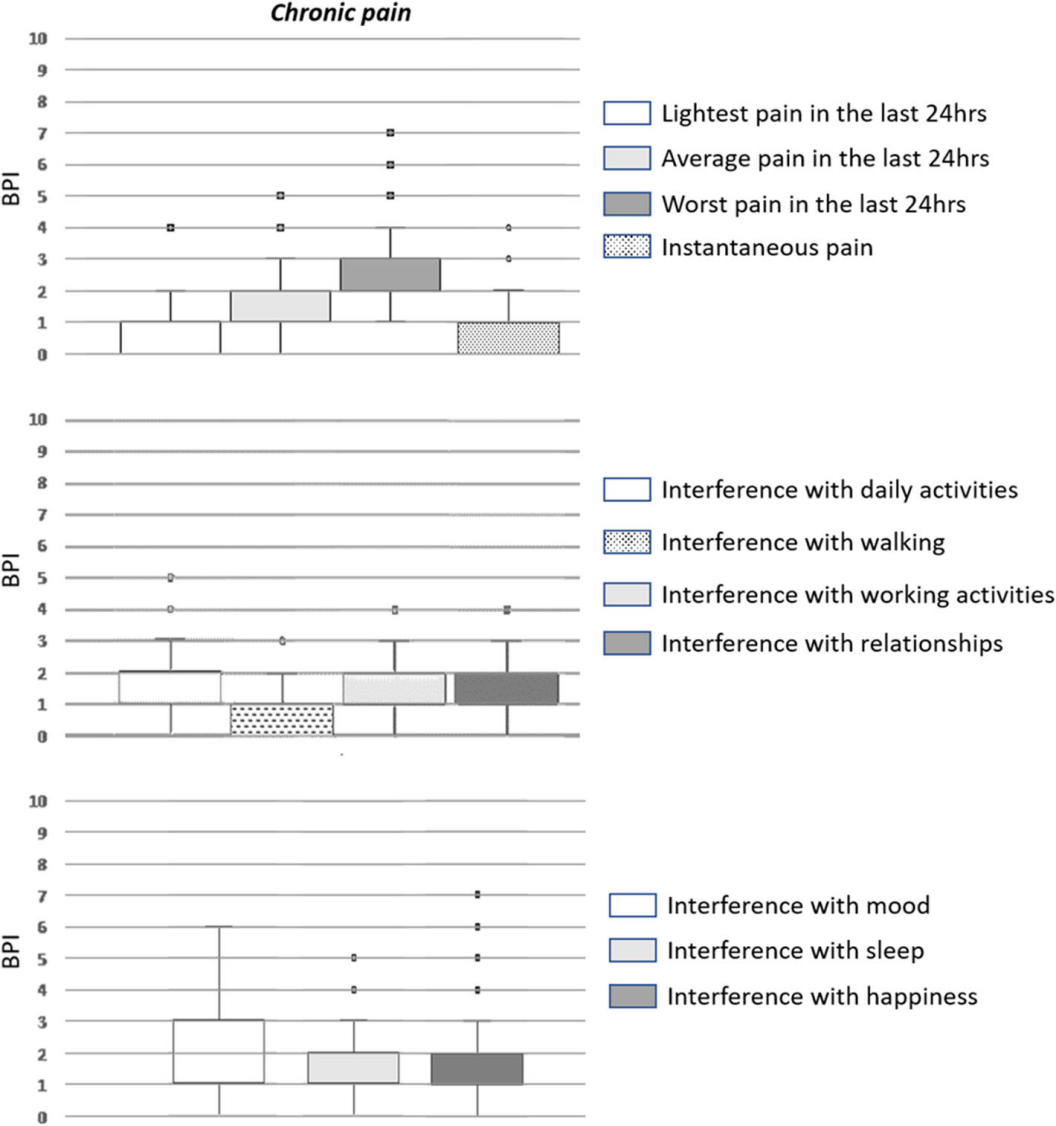
Intensity and impact of pain on patients’ quality of life as from the BPI questionnaire

All pain intensity and interference scores measured through the BPI questionnaire are presented in Fig. 2. BPI scores at 3 months are also reported in the Supplementary Table 1.

## Discussion

In this prospective, single centre, observational study performed in a large tertiary care teaching hospital, we have observed a 28% [95% CI 23.1–33.4%] incidence of CPBS in a cohort of female patients undergoing surgery for breast cancer. Axillary surgery, preoperative use of pain medications, and higher dynamic NRS values at 6 h postoperatively have been identified as independent predictors of CPBS.

Chronic pain is a very well-known complication of breast surgery (Alves Nogueira Fabro et al. 2012; De Oliveira et al. 2014; Tasmuth et al. 1996; Wang et al. 2018). Despite increasing recognition of chronic pain and the multidisciplinary efforts made to prevent this complication, the occurrence of persistent pain after breast surgery still remains high (Humble et al. 2018). The incidence of CPBS found in our cohort of patients (28%) falls within the range of values reported in previous studies (i.e., 25–60% (Gartner et al. 2009; Wang et al. 2018)). This wide variability in CPBS incidence mainly derives from the lack of a specific definition of CPBS, only recently provided by the IASP task force (Schug et al. 2019). The main significance of the present study lies in the fact that it provides estimates of the incidence of CPBS based on the IASP definition. Accordingly, in contrast with previous studies where CBPS was usually detected at 12–24 months postoperatively (Bell et al. 2014; Mejdahl et al. 2013), we have evaluated CPBS at 3 months follow-up. Furthermore, in order to avoid confounding factors or misinterpretation of signs or symptoms, each patient enrolled in this study was preoperatively taught how to correctly identify body areas (i.e., breast/anterolateral chest wall, axilla, ipsilateral arm) and symptoms of persisting postoperative pain, including neuropathic characteristics. Finally, follow-up assessment of pain at 3 months was carried out after the scheduled postoperative surgical and physiotherapy evaluations, so as to exclude other confounding causes of postoperative pain (e.g., seroma, hematoma, prosthesis infection, previous arm-shoulder pain). All these aspects have contributed to improve reliability of the estimated incidence of CPBS in our cohort of patients.

There is, however, no clear consensus about the definition of “high risk patients” for CPBS, thus making it difficult to identify those subjects who might benefit the most from careful, early pain assessment. A complex multidisciplinary, multiparametric approach involving also psychosocial and genetic factors has been suggested to perioperatively stratify patients and identify those at high risk of developing CPBS (Bortsov et al. 2020; James 2017). Nevertheless, because genotyping is expensive and usually unfeasible in routine clinical practice, strategies for risk stratification are currently based on the evaluation of those clinical and anamnestic, pre- and intraoperative predictors of CPBS that have already been identified in the literature. Given the observational nature of this study, it was not possible to explore genetic susceptibility in our cohort of patients. On the other hand, to the best of our knowledge, this is the first study evaluating selective pectoral muscle denervation as a potential risk factor for CPBS. Although denervation is a major risk factor for chronic neuropathic pain, none of the 19 patients undergoing selective pectoral muscle denervation for aesthetic purposes developed CPBS.

In contrast with previous studies, we have not observed an association between CPBS and patient age (Gartner et al. 2009; Vilholm et al. 2008), previous breast surgery (Vilholm et al. 2008), reconstructive surgery (Roth 2018), or implant-device placement (both above or below the pectoralis major muscle) (Wallace et al. 1996). Our study confirms that there is little evidence to suggest CPBS might be influenced by type of anaesthesia (Cho et al. 2013; Steyaert et al. 2016; Veevaete and Lavand’homme 2014). However, it should be recognised that most (81.8%) of the patients were treated with total intravenous anaesthesia thus making it difficult to reliably detect possible anaesthesia-related differences in CPBS. Similarly, the numbers of PECS blocks and local anaesthetics infiltration performed in this cohort are relatively small, thus potentially affecting our results. Although PECS block II has already been recognised as a protective factor for pain chronicisation (De Cassai et al. 2020), we have not observed this effect in our cohort. Furthermore, other techniques such as paravertebral blocks or epidural analgesia (Andreae and Andreae 2013) and other drugs such as ketamine or i.v. lidocaine (Crousier et al. 2008; Grigoras et al. 2012) known to have benefits in reducing chronic pain have not been used in this cohort of patients, and thus their role has not been explored in this study. The lack of information on the effects of locoregional anaesthesia and adjuvant analgesics in reducing CPBS should be recognised as a limitation of this study.

Independently associated factors predicting CPBS in our cohort of patients were long-term use of analgesics for pre-existing chronic pain, axillary surgery, and higher values of dynamic NRS at 6 h postoperatively.

Preoperative breast pain and/or pre-existing chronic pain before surgery are well-known risk factors for chronic postoperative pain, particularly for CPBS (Gartner et al. 2009; Wang et al. 2018). In this study, nearly a third of patients had preoperative pain, mainly low-back pain or migraine (52.6% and 44.2% of cases respectively). Preoperative painful conditions seem to predispose patients to the development of chronic post-surgical pain/CPBS, probably because the postoperative transition from acute to chronic pain is facilitated by long-term central and peripheral pain sensitisation (Ji et al. 2018). In this study, preoperative chronic pain was not found to be an independent risk factor for CPBS, except for the most severe cases requiring chronic analgesic drugs for pain management. As far as we are aware, this is the first study reporting a correlation between the use of preoperative painkillers and CPBS. In our cohort of patients, migraine (treated with paracetamol and NSAIDs), low-back pain (treated with paracetamol, NSAIDs, and corticosteroids), and chronic dental pain (treated with NSAIDs and gabapentin) were the main causes for long-term use of analgesics. No patients had pre-existing breast or axillary pain.

The association between axillary surgery and CPBS has already been reported in the literature. In particular, conclusions produced from a meta-analysis study including more than 19,000 patients have highlighted a 21% increased risk of developing CPBS in those subjects who underwent axillary surgery (Wang et al. 2018). In this study, axillary surgery was associated with a 93% increased risk of developing CPBS. A likely explanation is the use of different follow-up time frames for evaluating the correlation between axillary surgery and CPBS. Among the studies evidencing a correlation between axillary lymph node dissection (ALND) and CPBS, three explored this correlation within 6 months postoperatively (Alves Nogueira Fabro et al. 2012; Karen et al. 2002; De Oliveira et al. 2014), and the remaining two within 12 months (Meretoja et al. 2014; Thornton et al. 2013). A higher likelihood of surgery-related nerve injury—more frequent during ALND than sentinel node biopsy—has been advocated as the most probable cause of this association. Interestingly, differences between sentinel node biopsy and ALND as risk factors for CPBS have not emerged in our cohort of patients, probably because of the low rate of ALND included in this study (15.3% vs > 30% in other studies) (Wang et al. 2018). To the best of our knowledge, no study has so far evaluated the possible role of sentinel node biopsy as a risk factor for CPBS. Furthermore, it should be underlined that, beside the effects of single surgical procedures (as reported in Table 1), the combination of different procedures might also have a synergic/additive role in determining CPBS. This issue has not been explored in this study.

Higher dynamic NRS values at 6 h postoperatively play a role as independent factors to predict CPBS in our cohort of patients. Several studies have recognised presence and severity of acute postoperative pain as risk factors for postoperative chronic pain (Palotie et al. 2013; Tasmuth et al. 1996), particularly for CPBS (Fassoulaki et al. 2009). Our results suggest that one-point increase in dynamic NRS values corresponds to a 27% increased risk of developing CPBS. Median postoperative NRS values reported in this study, both at rest and during movement, suggest that adequate postoperative management of pain was generally achieved in the enrolled population. It is worth noting that patients who developed CPBS at 3 months exhibited higher NRS values than those who did not. In particular, statistically significant differences were observed at univariate analysis between CPBS and No-CPBS groups in terms of dynamic and static NRS values at 3 h, 6 h, 12 h, and 24 h postoperatively.

Pain intensity and pain interference with daily functions were assessed through the Italian validated version of the BPI questionnaire. This is a reliable, multidimensional tool for evaluating also non-oncological chronic pain and has already been used for CPBS (Bonezzi et al. 2002; De Oliveira et al. 2014). Analysis of BPI scores by item showed that chronic pain was of mild, moderate, and severe intensity in 96.5, 2.3, and 1.2% of cases, respectively. In 18 (20.9%) patients with CPBS, the median NRS values recorded during movement were greater than five, therefore reflecting moderate to severe chronic pain. These results are in line with those reported in the literature (Andersen and Kehlet 2013; Leysen et al. 2018; Peuckmann et al. 2009). Finally, according to the BPI questionnaire, CPBS had a profound impact on sleep quality, mood, and perception of happiness.

It is plausible that a number of limitations may have influenced the results of this study. First, this is a single-centre study carried out in a single teaching hospital. Second, our cohort of patients was quite small and therefore our findings need to be validated in a larger population. Also, the numbers of PECS blocks and local anaesthetics infiltration performed in our cohort of patients were too small to adequately address the impact of these procedures on CPBS. Third, we were not able to evaluate other factors such as psychological or genetic features as potential predictors of CPBS. A further limitation is that neither the numbers and levels of sentinel node biopsy nor the preservation vs cut of the intercostobrachial nerve were recorded. Also, we did not explore the effects of the combination of different surgical procedures in determining CPBS. Finally, we were not able to evaluate the effects of locoregional anaesthesia and adjuvant analgesics as well as the role of acupuncture and other non-pharmacologic analgesic therapies or interventional procedures in modulating CPBS.

## Conclusions

Chronic pain after breast surgery is a frequent complication that interferes with patients’ daily activities. In this study, evaluation of CPBS according to the IASP definition resulted in a 28% incidence at 3 months postoperatively. The possibility to early detect persistent pain, particularly in those patients at high-risk for CPBS, might help physicians to more effectively prevent pain chronicisation. In our cohort of adult surgical female patients, long-term use of analgesics for pre-existing chronic pain, axillary surgery, and higher dynamic NRS values at 6 h postoperatively were statistically associated with a higher risk of developing CPBS. A timely identification of high-risk patients might allow the implementation of proactive pharmacologic and non-pharmacologic approaches to prevent CPBS.

## Supplementary Information

**Additional file 1: Table S1**. BPI score in patients with pain at 3 months.

## Data Availability

The datasets analysed during the current study are available from the corresponding author on reasonable request.

## Abbreviations

CPBS: Chronic pain after breast surgery
IASP: International Association for the Study of Pain
NRS: Numerical rating scale
BPI: Brief Pain Inventory
PCA: Patient-controlled analgesia
ALND: Axillary lymph node dissection
