# Supplementary material for: Chronic pain after breast surgery: incidence, associated factors, and impact on quality of life, an observational prospective study
Source: Perioper Med (Lond). 2021 Feb 24;10:6. doi: 10.1186/s13741-021-00176-6 (PMC7903732; doi:10.1186/s13741-021-00176-6)
Supplement: Supplementary file 1 — Additional file 1: Table S1. BPI score in patients with pain at 3 months. [file 13741_2021_176_MOESM1_ESM.docx]

|  | BPI scores  (n=86) |
| --- | --- |
| **Pain Intensity** |  |
| Worst pain (Median,[Q1-Q3]) | 3[2,3] |
| Least pain (Median,[Q1-Q3]) | 1[1,1] |
| Average pain (Median,[Q1-Q3]) | 1[1,2] |
| Pain now (Median,[Q1-Q3]) | 0[0,1] |
| Dynamic NRS > 3 n. (%) | 18 (20,9%) |
| Dynamic NRS > 5 n. (%) | 3 (3.5%) |
| **Pain Interference** |  |
| General activity (Median,[Q1-Q3]) | 2[1,2] |
| Mood (Median,[Q1-Q3]) | 1[1,3] |
| Walking (Median,[Q1-Q3]) | 1[0,1] |
| Normal work (Median,[Q1-Q3]) | 1[1,2] |
| Social relation (Median,[Q1-Q3]) | 1[1,2] |
| Sleep (Median,[Q1-Q3]) | 1[1,2] |
| Enjoyment of life (Median,[Q1-Q3]) | 1[1,2] |
| **BPI Interference Score** |  |
| Low (1-4) n.(%) | 75(87,2%) |
| High (5-10) n.(%) | 11(12,8%) |

Table S1. BPI score in patients with pain at 3 months.
